# An adrenal incidentaloma caused by synchronous and isolated metastasis

**DOI:** 10.1002/ccr3.3996

**Published:** 2021-02-24

**Authors:** Koichiro Yamamoto, Kosuke Oka, Hiroyuki Honda, Kou Hasegawa, Yoshihisa Hanayama, Tomofumi Watanabe, Yusuke Tominaga, Atsushi Takamoto, Takayuki Hara, Fumio Otsuka

**Affiliations:** ^1^ Department of General Medicine Okayama University Graduate School of Medicine Dentistry and Pharmaceutical Sciences Okayama Japan; ^2^ Department of Urology Okayama University Graduate School of Medicine Dentistry and Pharmaceutical Sciences Okayama Japan; ^3^ Department of Nephrology, Rheumatology, Endocrinology and Metabolism Okayama University Graduate School of Medicine Dentistry and Pharmaceutical Sciences Okayama Japan

**Keywords:** adrenal metastasis, incidentaloma, lung cancer, oligometastasis

## Abstract

We report a patient with adrenal incidentaloma due to synchronous and isolated metastasis from lung cancer, which is a relatively rare condition. Close checkups for incidentaloma in oncologic patients are mandatory, leading to successful operation.

## Graphical abstract

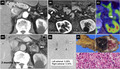

An 82‐year‐old male presented with a left adrenal incidentaloma (1 cm, Figure 1A) detected by contrast‐enhanced computed tomography (CE‐CT) that was performed for a thorough examination of primary lung cancer. Dynamic adrenal magnetic resonance imaging indicated that the adrenal tumor was compatible with adrenocortical adenoma (Figure 1B), and no metastatic lesion was detected in the whole body including the left adrenal gland by ^18^F‐positron emission tomography/CT (PET/CT) (Figure 1C). The patient had undergone surgery for lung cancer, and the tumor histopathology confirmed a diagnosis of adenocarcinoma. Three months later, CE‐CT showed an enlarged left adrenal tumor (3 cm, Figure 1D). Endocrinological workup for basal adrenal functions and scintigraphy examinations using ^131^I‐adosterol (Figure 1E) and ^123^I‐metaiodobenzylguanidine showed that adrenocortical and adrenomedullar functions were almost preserved and that the left adrenal tumor was nonfunctional. Laparoscopic adrenalectomy was performed for the left adrenal tumor. The resected adrenal had necrosis and bleeding in the tumor sections (4 cm, Figure 1F, *upper*), and histopathology of the tumor was compatible with metastasis of lung cancer (Figure 1F, *lower*), while the adjacent normal adrenal was morphologically preserved.

**FIGURE 1:**
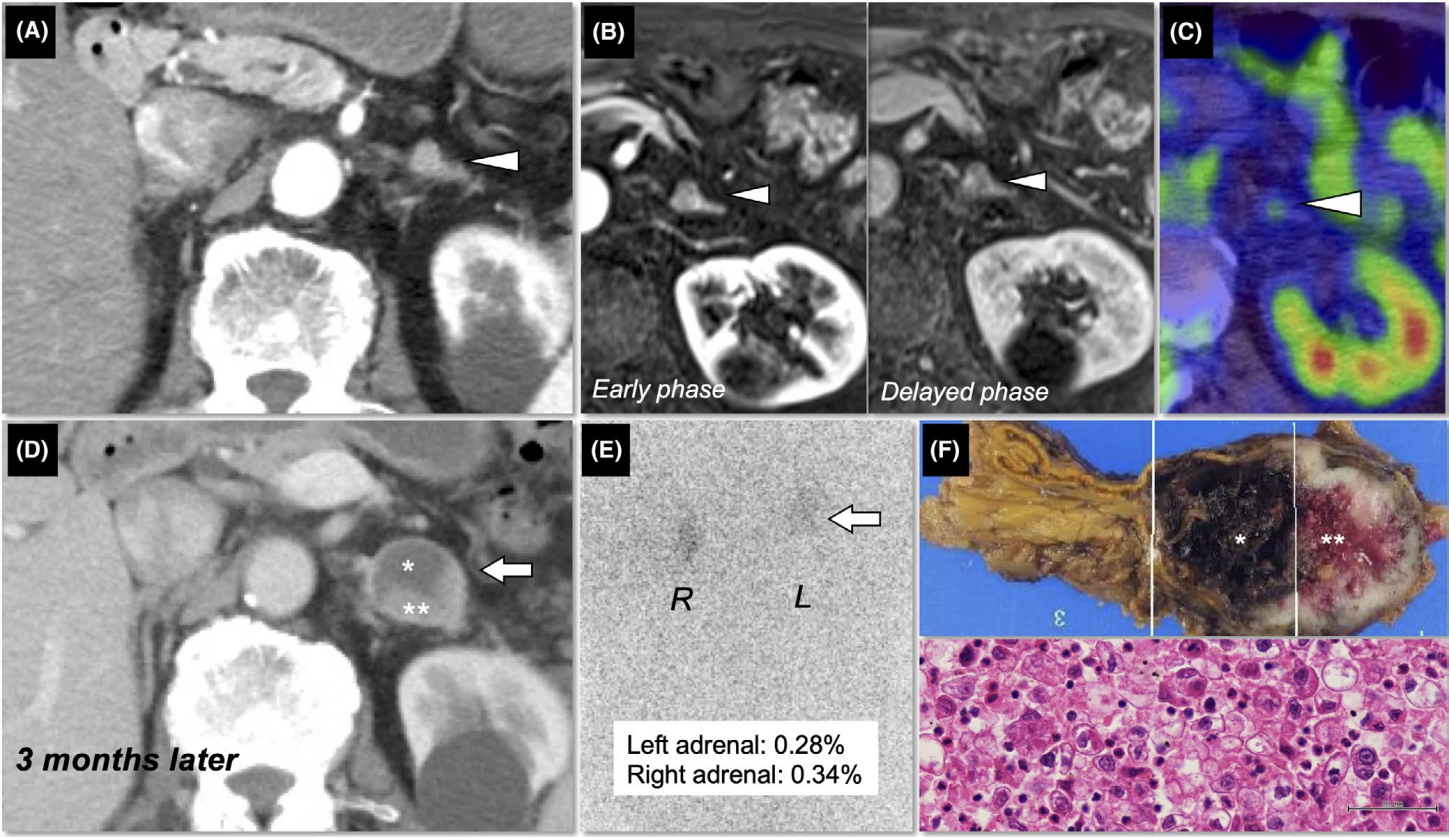
A, Contrast‐enhanced computed tomography (CE‐CT) revealed a left adrenal incidentaloma (1 cm, *arrowhead*). B, Dynamic adrenal magnetic resonance imaging suggested a left adrenal adenoma (*arrowheads*). C, ^18^F‐positron emission tomography/CT showed no metastasis including the left adrenal gland (*arrowheads*). D, Follow‐up CE‐CT revealed an enlarged left adrenal tumor (3 cm, *arrow*), suggesting that the tumor had necrosis (*) and bleeding (**). E, Scintigraphy examinations using ^131^I‐adosterol showed normal adrenal uptake. F, Macroscopic findings of the tumor section showed necrosis (*) and bleeding (**) (*upper*), and tumor histopathology was consistent with lung cancer metastasis (*lower*)

The occurrence rate of adrenal metastases in patients with lung carcinoma is 35%,^1^ whereas the incidence of isolated adrenal metastases is relatively low.^2^ Prognosis of patients treated with adrenalectomy for synchronous and isolated adrenal metastasis from non‐small‐cell lung cancer, as in our case, is poor,the median survival rate is 1 year.^2^ Oncologic patients, such as patients with lung cancer, breast cancer, or malignant melanoma, should be carefully monitored for secondary adrenal incidentaloma, even in cases in which metastasis was not initially detected by PET/CT.

## CONFLICT OF INTEREST

The authors declare no conflicts of interest.

## AUTHOR CONTRIBUTIONS

KY: wrote the first draft and managed all of the submission process. KO, HH, KH, YH, TW, YT, AT, and TH: contributed to the clinical management of the patient. FO: organized the manuscript.

## ETHICAL APPROVAL

Informed consent was obtained from the patient to publish this case report.
